# Towards a Better Understanding of Sickness Absence in Adolescence: A Qualitative Study among Dutch Intermediate Vocational Education Students

**DOI:** 10.1155/2017/1064307

**Published:** 2017-05-09

**Authors:** Yvonne T. M. Vanneste, Frans J. M. Feron, Marlieke A. W. van Mook, Angelique de Rijk

**Affiliations:** ^1^Regional Public Health Service West Brabant, Tilburg, Netherlands; ^2^Faculty of Social and Behavioural Sciences, Tranzo, Tilburg University, Tilburg, Netherlands; ^3^Department of Social Medicine, Faculty of Health, Medicine and Life Sciences, CAPHRI, Maastricht University, Maastricht, Netherlands

## Abstract

An adequate approach to sickness absence can reduce school dropout which is a major problem in Intermediate Vocational Education (IVE). This practice-based study explores the sickness absence reasons and factors influencing reporting the sickness, from a student's perspective. Semistructured interviews were held until saturation. Data were collected and analysed by a multidisciplinary research team including youth health care physicians working with IVE students. The results show that, according to the students, reasons for sickness reporting were health-related or related to problems at home or in school. Students view their sickness absence as necessity, as asking for understanding, or as pardonable. Their views depended on (1) the perception of medical legitimacy, (2) feeling able to take their own responsibility, (3) feeling being taken seriously at school, and (4) the perception that the sickness reporting procedure at school is anonymous and easy. In conclusion, reporting sickness seems more a reaction to a necessity or opportunity than the result of a conscious decision-making process. Personalizing the sickness reporting procedures and demonstrating interest rather than control while discussing the sickness absence with the individual IVE student might very well affect their sickness absence levels.

## 1. Introduction

School absenteeism is a major problem, both for the individual and for society. It may lead to a lower level of education and school dropout [1–3]. Low educational level and school dropout are both strongly associated with increased risk behaviour [4, 5], a higher prevalence of chronic health issues and common mental problems [6, 7], higher mortality rates [8–12], and an increased risk of social failure and delinquency [13, 14]. In the Netherlands, the prevalence of school dropout is highest at schools for Intermediate Vocational Education (abbreviated as IVE; ISCED 3C) [15]. In the school year 2011-2012, 71.5% of all school dropouts left school from IVE [16]. In particular, those dropouts with health problems (including mental health problems) appear to be subsequently less successful in their individual career development and lives [17, 18].

School absenteeism due to sickness reporting, also called sickness absence, is as common as truancy, or even more prevalent [19–22]. In the Netherlands, contrasting with the widespread implementation of sickness absenteeism prevention and guidance in the workplace, there are no interventions and agencies addressing sickness absence among students. Recently, the intervention “Medical Advice for Sick-reported Students” (MASS) has been developed to address sickness absence in prevocational secondary and IVE schools [23–26]. MASS consists of a standardized approach by which schools, in direct collaboration with youth health care physicians, act upon student's sickness reporting, followed by making and monitoring a management plan to optimize students' health and maximize students' participation in school activities. MASS is now tested in a limited number of schools and nominated to be implemented at a larger scale. However, the effects of MASS might improve, if communication with the students incorporates a better understanding of their perspective on the sickness absence.

In the Netherlands, the IVE student is the key actor as he is required to report oneself sick. There are indications that what students report varies from what really happened. Derriks et al. [27] studied the reporting of absenteeism by IVE students and found that almost 40% of the students stated that they do not report their absence because it is rather easy to be absent from school unnoticed; 20% stated that if reporting their absence, they do not tell the real reason for absence. Recently, de Kroon et al. [28] found that students were much more convinced than the other stakeholders that sickness absence has most often medical reasons. When acting upon the student's sickness reporting, as requested from schools using MASS, it therefore seems to be important to gain a better understanding of why and how IVE students report sick.

**Figure 1:**
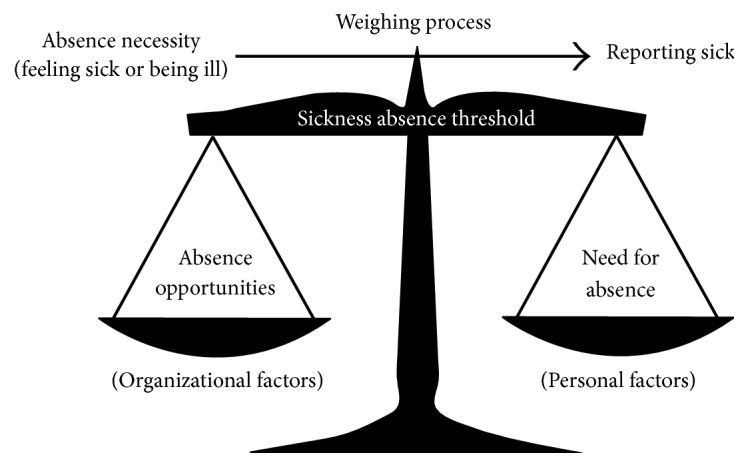
A model with aspects of the individual and workplace situation that are taken into account before reporting sick. Adapted from the decision model (Veerman, 1993).

Studies and models from occupational health are informative for this topic for two reasons. First, during work placements these students will also be expected to function as employees. Second, given their age, one should be able to appeal to their autonomy. Veerman's model [29] is one of the many models for occupational absenteeism due to sickness [30]. The Veerman model (Figure 1) is a decisional model [30] and distinguishes different aspects of the individual and workplace situation that are taken into account before reporting sick. The model explains that “absence necessity” (i.e., feeling sick or being ill) is moderated by a “sickness absence threshold.” This threshold is in turn influenced by “absence opportunities” and “need for absence” (affected inversely by need to be present). “Absence opportunities” refer to organizational factors and legislation that allow for absence. “Need for absence” refers to the subjective values of the job and how much an employee wants to report sick due to the job. This model represents a two-stage process: there must be an absence necessity before a process of weighing pros and cons starts.

This model can be used as a starting point to study IVE student perspectives on their sickness absence. However, the difference in age between these students and employees and the difference in relationships that exists between school and student versus that between employer and employee are elements for consideration.

The research was thus inspired by observations in practice and by theoretical notions on sickness absence among adult employees. The purpose of this study was to gain insight into the specific dynamics of IVE students' sickness reporting according to students themselves. This bottom-up practice-based knowledge of sickness absence in IVE students could make prevention and management of sickness absence among this group more effective [31].

The research questions for this study are as follows: (1) What are, according to the students, their reasons for sickness absence? (2) Which factors influence their sickness reporting according to the students? (3) To what extent does Veerman's model apply to the sickness absence of IVE students?

## 2. Methods

Qualitative research methods are useful when exploring stakeholders' perceptions [32]. To find answers to our research questions and to obtain proper insight into the experiences and perceptions of the students, in-depth individual semistructured interviewing was selected as the best method of data gathering [32, 33]. Data were collected between February and June 2014 in the North Brabant region in the southwest of the Netherlands. In order to obtain as many different views as possible, purposive sampling was used. Students who were especially knowledgeable about sickness absence and able to communicate their experiences and opinions in an expressive and reflective way were identified and selected [33]. Participants were approached until data saturation had been reached, meaning that no new data and concepts were identified [34].

### 2.1. Study Participants

To be included, students had to attend IVE and had to have experienced sickness absence. In the Netherlands, IVE has four educational levels. Level 4 is the highest level and gives access to institutes for Higher Vocational Education and Training [15]. Students were first asked to participate in the study by a care coordinator of the school. It appeared to be difficult to recruit students this way: only four students were willing to be interviewed. Then, by using the “chain-referral sampling” method [35], the next 12 students could be recruited. Because, in the beginning, only students who were receiving education at level 4 were willing to participate in the study, participation was eventually limited to this level. To obtain as many different views as possible, students with different background characteristics (gender, age, etc.) were searched for. Students to be interviewed were added until no additional new concepts were identified; saturation occurred after fourteen interviews and was confirmed through two final interviews. The participant characteristics are presented in Table 1.

### 2.2. Ethical Considerations

All procedures were in accordance with the ethical standards of the institutional and national research committee and with the 1964 Helsinki declaration and its later amendments or comparable ethical standards. For both ethical and quality reasons, the study was conducted according to the Code of Conduct for Medical Research of the Council of the Dutch Federation of Medical Scientific Societies. Students were informed by letter about the purpose of the research, the anonymously processed data analysis, and the use of the information received, exclusively for research and in which confidentiality was guaranteed. They were made aware that they were free to refuse to participate at any time before, during, and after the interview. They were given three days for reflection before an appointment had to be made. The interview was only initiated upon receipt of written consent. In cases of being under the age of 18 years, written informed consent of the parents was also received.

### 2.3. Interview Procedure

The questions were broad, open-ended, and nondirective. The interview topic list was inspired by Veerman's model and the experience of youth health care physicians and career counsellors in IVE. It included questions such as (a) why the student reported sick from school (from their perspective), followed by questions such as (b) what is the sickness reporting procedure in your school, (c) when and how do you report sick, (d) what happens after that, (e) who was involved in your sickness reporting, (f) how do your classmates deal with sickness reporting, and (g) from whom do you expect support after reporting sick? At the end of the interview the student was offered a conversation with the career counsellor or the youth health care physician. None of the respondents actually made use of this offer. The audio-recorded interviews lasted around 45 minutes, and were transcribed verbatim and anonymized for further analysis.

### 2.4. Analysis

An inductive thematic analysis was carried out [32] by structuring data, initial coding, categorizing experiences and perceptions, and identifying themes and their interrelations. In the multidisciplinary research team, different interpretations were discussed and refined until agreement was reached.

To gain enhanced insight into the sickness absence, two researchers were youth health care physicians working in different practices (YV and FF), and one researcher was a sickness absence researcher (AdR). Several intermediate analyses were applied to check whether all topics had been covered. All data (transcripts, coding tree, and findings) was available for inspection by the coauthors during the research. In addition, a three-hour group meeting took place, in which a multidisciplinary team of career counsellors, youth health care physicians, and school attendance officers was asked whether they considered the findings to be clear, understandable, and logical. On the basis of this meeting the analysis was refined.

### 2.5. Availability of Supporting Data

The study data is stored by the Faculty of Social and Behavioural Sciences, Tranzo, Tilburg University, in The Netherlands following the institution's data management policies. Data are fully available without restriction.

## 3. Results

To begin with, three ways are found in which students view their absent periods: they regard their absence as (1) necessity, (2) asking for understanding, or (3) pardonable. These three views can be understood by varied background experiences: the degree to which they feel able to take responsibility for themselves; the degree to which the sick-reporting procedure at school is experienced as anonymous and (too) easy; and the degree to which they feel themselves to be taken seriously by the school.

### 3.1. How Students Regard Their Absence

When asking for the reason for sickness absence, all the students spontaneously gave a sort of “retrospective justification” for their sickness absence. Three views could be distinguished here.

A first view was that of absence as necessity. The students found their sickness absence necessary and legitimate. They indicated that they report sick because they are sick or have physical complaints.

I was sick two years ago. A problem with my nerves. And I missed eight months. (N., 20 y.)

I had flu and a temperature. Yes, I did try, but it wasn't on. So then I just went home. (D., 20 y.)

A second view was that of absence as asking for understanding. The students excused themselves and asked for understanding for their sickness absence, because they were at a loss or were confused and upset. They expected that reporting sick will help them to feel better. They reported that psychological complaints and problems at home or at school made them report sick.

I was much too occupied with myself, so then what's the point of being at school?…And I explained that. I think I can expect allowance to be made for this. (D., 19 y.)

If I need to calm down I report sick. (H., 24 y.)

There is still a rather unpleasant atmosphere in the class… it puts very many students off, and then they sometimes simply report sick. (T., 19 y.)

A third view was absence as pardonable. Then, the students regarded their reporting sick as pardonable and believed that school should not constrain them. Some of them blamed others for their sickness reporting and said that they occasionally reported sick because of reasons such as the following: other students reported also to go home, have free periods during the day, and complained about the noninspiring method of teaching.

I think that many students think that a little bit of absence during school time is not really a problem. (F., 18 y.)

If coming to school is just a waste of time, then you can expect people to think up an excuse for not having to come any more. Anyone would do that if they did not find a lesson interesting. You know, some more interesting and active lessons, those are more appealing. (W., 19 y.)

Some participants also commented on their fellow students reporting this type of sickness absence. They indicated that some students report sick because of excuses such as the following: being too tired after the weekend, not/no longer feeling like going to school, being too lazy to get out of bed, or having something else to do.

Some are not really motivated, and they go out in the evening or find football more interesting. Others might prefer going for a joint. (R., 18 y.)

Views can be mixed within one student.

If I need to calm down I report sick, or if I'm really sick, or if I've got too much going on in my head. (H., 24 y.)

These three views expressed variations in taking responsibility, valuing the school sick-reporting procedure, and feeling treated seriously, which is explained next.

### 3.2. The Degree to Which They Feel Able to Take Responsibility

Some students said they were quite able to take responsibility. Other students indicated that they were still learning to take responsibility, because they were still maturing into adulthood.

…I live alone and take responsibility for everything myself. (H., 24 y.)

I think that those who are older should be able to imagine what it's like to be young. That things can be quite difficult for us. Or how to cope, because sometimes I simply have no idea. We have to learn it all. (D., 19 y.)

Students said that their parents could help them with this. Some students indicated that their parents had little involvement with school, while other students said that their parents did involve themselves in school matters.

Even if I were 18 I would still get my parents to report me sick. So I couldn't misuse it … Yes, I also prefer them to know what's going on. (M., 17 y.)

My parents were always on my back. If they hadn't done that it would have been much worse, I think. (W., 19 y.)

My parents have left me free to do what I want. (T., 23 y.)

Some students said that they spoke openly at home about their sickness absence, while others kept it from their parents.

I'm honest with my mother, but she says it's your own affair as long as you get through. (J., 22 y.)

I did not say that I went home sick. I was afraid that they would keep on at me if I told them, and I do not want that. If I were honest about it they wouldn't support me, and I suppose that's right really. By not telling my parents I avoided extra stress. (T., 23 y.)

Students also indicated that the school could help them by more teaching to take responsibility. In order to do so, school should acknowledge that the student is not yet able to take full responsibility, and they should present clear consequences to the sickness absence.

I think, so help us with it, but that's not how I see things here at school. (D., 19 y.)

They do not necessarily have to do anything specific, but I hope that the school can at least show some understanding. (L., 19 y.)

There would be a lot fewer students absent if the school took more action against excessive absence. (W., 19 y.)

The students expressed that when on work placement, they feel a greater obligation to turn up.

I do not make plans to meet up during work placement time, because I find that a bit more serious than school, because then you're actually in a company and it's not really on to say each week that you've got something else to do. And the companies also quickly get fed up with it and you have to look for another company. It's not easy to skip work placement, and I wouldn't recommend it to anyone. (F., 18 y.)

Taking responsibility is one variation, but students also reported that variation in procedures at school affected their sickness absence.

### 3.3. The Degree to Which the Sick-Reporting Procedure at School Is Perceived as Anonymous and Too Easy

The students explained how one can call in sick and whether parents are involved or not. Most of the students who had to report sick digitally indicated that this was too easy, meaning that they were ready to call in sick more easily.

I think that people are quick to report sick when they can report sick online… (so easy)…. (J., 22 y.)

The students also indicated that they miss a personal way of reporting sick, for example, to the career counsellor. They said that the person at the other end of the line could support by allowing students to get the story off their chest and to know straight away what the school thinks about it.

… While you're on the phone you have to talk to someone and tell them what the problem is. They may well come to the opinion that you are not really sick … But the main thing is that you can tell about what's really the matter. Then at least you hear their voice. At present you have no idea what they think about your sickness absence… I would prefer a more personal way of reporting sick. (F., 18 y.)

At the same time, the students indicated that they have no idea about the function of career counsellors and whether they could rely on them. They said that the talks they had with their career counsellor could be more strongly focused on individual guidance.

… So I do not know whether I can count on the career counsellor if there is something wrong at home, I really couldn't say…. (F., 18 y.)

… I now have a career counsellor, and last year he really said something wrong. He told me, you must keep school and private life separate. Well sorry, I find that really stupid. There is a reason for having a career counsellor, is not there? You simply have a talk, and then they ask how things are going. Are you getting good marks? Okay, good, send in the next one. Surely it's not only about your marks? Isn't it also about your development as a person? (D., 19 y.)

Other students indicated that they did not feel their relationship with their career counsellor was confidential enough for them to wish to discuss their problems with him or her.

That Mrs X is very pleasant but she really gives the impression that she thinks you're stupid. Everyone says that you've got someone you can go to and that's Mrs X. Yeah, yeah, it's not going to be her, that's the last person I would go to with my problems. (T., 19 y.)

Besides variation in procedures, there was also variation in the experience to which the school took the student seriously.

### 3.4. The Degree to Which They Feel Themselves to Be Taken Seriously by the School

The students explained that they could not be expected to take all responsibility themselves but this should not lead to not being taken seriously by the school. Schools appeared to react in many different ways to a sickness reporting. Some students said that the reaction took a long time and sometimes never even took place. They would like the school to inquire about the reason for sickness absence.

…No, they did not ask about it… a classmate was genuinely sick, but they did not really ask after her. They never asked how she was. So I think, that's pretty bad…. (M., 19 y.)

If you really report sick frequently then I would expect them to want to talk to you about it: why do you report sick so often, and how can I help you? Do you enjoy school or not? What do not you like, and what is it that you do like? Is the course right for you? Is something else the problem, are there problems at home? You look into the background of your students, so that you, as career counsellor, can watch out for things or give guidance. (L., 19 y.)

One of the students claimed to experience too much checking up after reporting sick.

So they really checked up to see whether we were really sick … Just as if they were always suspicious that you are pretending to be sick, or playing truant, or that sort of thing. (J., 21 y.)

Students reported that if they were taken seriously they would make more effort to avoid absence.

There are really quite a few who are away a lot. I have the idea that it's really badly handled. Now and again they're called to account, but that's it… If the system were different, then I would be better myself and would put more effort into acting differently. When it's so easy, you find it easier to do it yourself. And it does not work like this in real life, so why deal with it this way here? (F., 18 y.)

The students noted that the ease with which they could stay away from school hindered them in taking their own responsibility for reporting sick.

## 4. Discussion

The purpose of this study was to gain insight into the specific dynamics of IVE students' sickness reporting according to students themselves. In total 16 Dutch students at the highest level of IVE were interviewed. They were asked for reasons for and factors that, in their opinion, influence their sickness reporting. To gain enhanced insight into and understanding of sickness absence among IVE students and into how public health practice can address sickness absence more effectively, the research was carried out as a practice-based study in close collaboration with youth health care physicians and by a multidisciplinary research team. The implications of the results for the practice of youth health care and public health and the extent to which the results fit with the theoretical model from occupational medicine [29] will be discussed.

**Figure 2:**
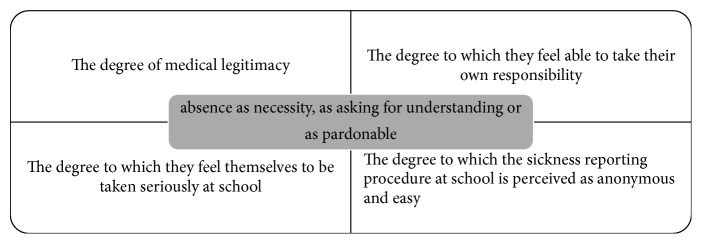
Schematic representation of the relationship between the way students perceive their sickness absence and the background experiences involved, according to the students.

All students spontaneously gave a “retrospective justification” for their sickness absence. Three views on sickness absence could be distinguished: as necessity, as asking for understanding, or as pardonable. These views could be understood by the following background experiences: the degree to which they feel able to take their own responsibility, the degree to which the sickness reporting procedure at school is perceived as anonymous and easy, and the degree to which they feel themselves to be taken seriously at school.  Figure 2 presents a conceptual model schematically illustrating the relationship between the students' view on sickness absence and the aforementioned factors.

The reasons for sickness absence given by the students themselves varied from “really too ill to go to school” to “got something else to do,” indicating that the degree of medical legitimacy differs. Reasons for sickness absence among IVE students were not only sickness but also mental health problems caused by the home or class situation, together with the behaviour of peers, the timetable, and the content of the lessons. Reasons as “too tired or lazy” given by students to explain away their absence may be linked to school disengagement which may be strengthened by the feeling of not being taken seriously by school and by anonymous sickness reporting procedures. School absenteeism can be a result of school disengagement. In addition, students are often sanctioned as response to absenteeism. This might contribute to further negative perceptions about investing in school and leading to increased school disengagement and school absence [36, 37]. Students indicating that they report sick because they “have something else to do” may not have the right attitude towards school. In such cases it will probably be important to link clear consequences to (too much) sickness absence. However, a wrong choice of study might also cause “not feeling like school any more.” And “too tired or lazy” could hide a lifestyle problem (addiction, lack of exercise, poor diet, etc.). It is thus important to listen to the whole individual story behind the sickness absence report, but such response is now often lacking. Further, sickness absence might also be prevented by general measures as promoting a good atmosphere in the class and well-scheduled lessons matching the capabilities and interests of students.

The decision model of Veerman [29] assumes an absence necessity as a motive for the absence, after which a weighing of pros and cons is made by taking personal factors that indicate the need for absence and organizational factors that indicate the opportunity for absence into account. This results in a conscious, well-balanced decision to report absence or not. A weighing process in the case of the IVE students seems to be lacking. Even though there is an absence necessity (e.g., a health problem), the sickness report appears to be more an ad hoc decision. If it all gets too much for students, they call in sick without feeling able to oversee the consequences. And this occurs, according to the students, more easily if the sickness reporting procedure is impersonal and too easy. Their “retrospective justification” implies also that the consequences do not play a conscious role when reporting sick. Reporting sick is an immediate reaction to a need, a desire, or an opportunity rather than a process of consideration. This might be explained by the students' state of development. When considering something, one needs to be able to form a view of long-term consequences of decisions. At the age of these IVE students (16 to 26) the prefrontal cortex, responsible for planning and decision-making, is often not yet fully matured [38]. In line with this, the degree to which, in their own opinion, students feel up to taking responsibility varied across students. Although the students suggest that their parents could help them in learning how to take responsibility, the interviews show that this cannot be realized on the same level for every student. Parents' involvement with school and the effectiveness of this involvement according to the student varied largely between students. While one student spoke about his sickness absence at home, another concealed it, out of fear of causing trouble and stress. Research indeed showed that the withholding of information by students, especially in cases of questionable acts, was primarily due to concerns about parental disapproval [39]. Precisely at this age young people are trying to establish their independence of home [40, 41], and parental interference is not always appropriate. This can explain the differences and ambiguity concerning the help wanted. Whereas for students the goal of adolescence is to gain independence and to establish an identity; the challenge for parents is to correctly assess the degree to which their child needs guidance in this process [42].

The students in this study do have concrete ideas on how the school can teach them taking more responsibility. In the first place the students asked the school to show some understanding for the fact that they are “learning.” They consider that linking consequences to sickness absence and clear communication about these may help them to (learn to) take more responsibility. More personal reactions on their reporting sick—directly and afterwards—could help in learning to take responsibility because students then get the chance to explain their situation. Students wish to know an adult's opinion about it if they feel sincere interest and confidence. This will teach them making more proper judgements. At the same time the students indicate that they do not have a clear picture of the value of a career counsellor and that a confidential relationship is important. This is also found in literature: students are often sanctioned by school staff in response to school absenteeism and, unfortunately, this contributes to further negative perceptions about investing in school, thus leading to school dropout [36].

The students indicate that their experience with reporting sick on previous occasions plays a role in case of a new occurrence. Factors such as easiness, anonymity, and the lack of a reaction to the sickness report by the school lower the threshold to report sick.

The practice-based character of the study is an important strength. The research questions, data collection, and data analysis were inspired by both practice and research. The results are relevant for practice and also have an added value to the theoretical modelling of sickness absence in IVE students. This mix of research and practice was also reflected in the multidisciplinary research team. A limitation of this study is that the results only apply to students at the highest level of IVE. It might be that other underlying problems play a role in sickness absence among the students from the three lower levels, including lack of understanding of procedures, school failure, and other expressions of lower cognitive abilities. Also, the results might be limited to Dutch IVE schools, although the results did not seem to be linked to specific school characteristics.

For practice, it is important to personalize the sickness reporting procedures in schools as much as possible. Only then, students will feel being taken seriously, an opening to address the underlying reasons will be given, and students are supported in the process of taking more responsibility regarding their sickness absence. When demonstrating interest rather than control, school will be more easily given access to the student's own view of sickness absence, thus preventing disengagement from school. Connecting to the student's view is also expected to facilitate organizing effective care and guidance.

Regarding the theoretical model, reporting sick seems in this student population more a reaction to a necessity or opportunity than the result of a conscious decision-making process. Regarding further research, it is recommended to also study the issue from school perspective in order to get a more comprehensive understanding of sickness absence in IVE students. Furthermore, it is recommended to study if and how positive reinforcement of presence instead of sanctioning of absence could increase school attendance rates. This is expected to increase the effectiveness of prevention and management of sickness absence for this student group.

## Figures and Tables

**Table 1:** Overview of students' characteristics.

	Gender	Age	Living at home	Ethnicity	School	Sick reporting system	Subject of education
N.	Male	20	Yes	Native	1	Digital	Architecture
H.	Male	24	No	Immigrant^1^	1	Digital	Architecture
D.	Female	20	Yes	Native	2	By phone	Legal services
D.	Female	19	Yes	Native	2	By phone	Legal services
R.	Male	18	Yes	Native	3	By phone	Entrepreneurship
M.	Female	17	Yes	Native	2	By phone	Legal services
J.	Male	19	Yes	Native	1	Digital	Architecture
F.	Male	18	Yes	Native	1	Digital	Architecture
L.	Female	17	Yes	Native	2	By phone	Legal services
T.	Male	19	Yes	Native	3	By phone	Entrepreneurship
L.	Female	19	Yes	Native	4	By phone	Teaching assistant
J.	Male	21	Yes	Native	5	By phone	Leisure
M.	Female	19	Yes	Native	4	By phone	Social care
T.	Male	23	Yes	Native	5	By phone	Branch manager
W.	Female	19	Yes	Native	6	By phone	Veterinary assistant
J.	Male	22	Yes	Native	7	Digital	Sports

^1^Second-generation immigrant.
